# Potential Role of Exosomes in Mending a Broken Heart: Nanoshuttles Propelling Future Clinical Therapeutics Forward

**DOI:** 10.1155/2017/5785436

**Published:** 2017-10-15

**Authors:** Julie A. Dougherty, Muhamad Mergaye, Naresh Kumar, Chun-An Chen, Mark G. Angelos, Mahmood Khan

**Affiliations:** Department of Emergency Medicine, College of Medicine, The Davis Heart and Lung Research Institute, The Ohio State University Wexner Medical Center, Columbus, OH 43210, USA

## Abstract

Stem cell transplantation therapy is a promising adjunct for regenerating damaged heart tissue; however, only modest improvements in cardiac function have been observed due to poor survival of transplanted cells in the ischemic heart. Therefore, there remains an unmet need for therapies that can aid in attenuating cardiac damage. Recent studies have demonstrated that exosomes released by stem cells could serve as a potential cell-free therapeutic for cardiac repair. These exosomes/nanoshuttles, once thought to be merely a method of waste disposal, have been shown to play a crucial role in physiological functions including short- and long-distance intercellular communication. In this review, we have summarized studies demonstrating the potential role of exosomes in improving cardiac function, attenuating cardiac fibrosis, stimulating angiogenesis, and modulating miRNA expression. Furthermore, exosomes carry an important cargo of miRNAs and proteins that could play an important role as a diagnostic marker for cardiovascular disease post-myocardial infarction. Although there is promising evidence from preclinical studies that exosomes released by stem cells could serve as a potential cell-free therapeutic for myocardial repair, there are several challenges that need to be addressed before exosomes could be fully utilized as off-the-shelf therapeutics for cardiac repair.

## 1. Introduction

Cardiovascular disease (CVD) accounted for 30.8% of all deaths in the United States in 2014, with one person dying from CVD every 40 seconds [1]. More than half of all cardiovascular events in men and women under the age of 75 years are caused by coronary heart disease (CHD) [2], which includes myocardial infarction (MI). Furthermore, for patients over 45 years of age, 36% of men and 47% of women will die within 5 years after their first MI [1]. The primary treatments for CHD include antihypertensive and cholesterol-lowering drugs and surgical interventions including stents and bypass, all of which aim to prevent recurrence of MI or slow down heart failure. Unfortunately, these strategies do not address the issue of post-MI scar formation which often leads to progressive heart failure and eventually death. Research has been ongoing to prevent scar formation and improve cardiac function post-MI by encouraging cardiomyocyte regeneration in the infarct area.

Transplantation of stem cells is a viable therapeutic approach as the adult human heart has a very limited capacity for innate cardiac regeneration [3]. The potential of certain stem cells for multilineage differentiation provided the theoretical basis for their use in direct regeneration of injured cardiac tissue [4–6]. More recently, interest in using stem cells for cardiac repair was piqued with the discovery of induced-pluripotent stem cells [7] and subsequent derivation of functional cardiomyocytes [8], which could directly regenerate the injured tissue. However, theory has not been easily translated into practice as transplantation of stem cells has yielded limited success due to poor engraftment of stem cells in the ischemic heart [9, 10]. Interestingly, posttransplantation cardiac function improves even though the number of surviving transplanted cells present is very low [9, 10] and increased capillary density has been observed even though direct differentiation of the transplanted cells is lacking [11]. As such evidence is pointing towards a greater role of the paracrine signaling potential of transplanted cells, the key tenet in restoring cardiac function after MI may lie in providing the appropriate signaling events to initiate cardiac repair mechanisms. Recently, exosomes have emerged as a novel cellular signaling mechanism and can provide active molecules to target cells to aid in responding to stress. Delivering exosomes to damaged tissues to convey beneficial signals is of particular interest in cardiac regenerative medicine [12, 13]. Endogenous post-MI cardiac repair is inefficient and results in a maladaptive response that ultimately leads to heart failure [14]. Stimulation of endogenous remodeling and increasing local angiogenesis to support cardiomyocyte function and improve heart function is paramount to improving clinical outcomes for ischemic heart disease, and exosomes have the potential to fulfill this need.

## 2. Background on Exosomes

Exosomes are membrane-bound vesicles secreted by many cell types containing proteins [15], lipids [16], and nucleic acid [17–19]. Since numerous types of extracellular vesicles (EV) have been described, certain criteria exist to classify EVs as exosomes [20]. Exosomes are formed by inward budding of multivesicular endosomes, where molecules are packaged and stored [21] and later fuse with the plasma membrane for extracellular secretion. Exosomes are characterized by their size (40–100 nm) [22], which along with other physical properties allows for simple separation from debris released by cells and other types of vesicles [23]. Exosomes, once thought to merely be vehicles for waste disposal [24, 25], are now considered to play a critical role in intercellular communication and thus provoked fervent interest in understanding this novel function. Proteomic analyses unveiled that exosomes contain distinct proteins [26–28], which distinguish them from membrane vesicles released by apoptotic cells [21]. These nanoshuttles relay information from their cellular microenvironment to near and distant cells to signal necessary changes to deal with stressors.

## 3. Exosome Uptake by Target Cells

The lipid bilayer of exosomes protects the protein and nucleic acid contents, allowing them to persist in the extracellular environment. Exosome uptake by target cells has been shown to occur via myriad mechanisms in cell culture experiments, with uptake depending on the source cells, target cells, and the microenvironment. Exosomal uptake can take place by endocytosis; however, the endocytic pathway has multiple mechanisms for uptake. Fitzner et al. labeled purified exosomes with PKH67 and incubated them with mouse oligodendrocytes; they observed colocalization of exosomes with Lamp1, a marker of late endosomes, indicating that the exosomes had been internalized by an endocytic pathway [29]. Similarly, Tian et al. utilized labeled exosomes from rat pheochromocytoma cells and studied their uptake by cardiomyoblast cells pretreated with various pharmacological and chemical agents against specific mechanisms of endocytosis. Results from this study concluded that exosome uptake occurred via clathrin-mediated endocytosis and macropinocytosis [30]. Furthermore, Svensson et al. studied the efficiency of uptake of exosomes derived from human primary glioblastoma cells. Experiments determined the mechanism of uptake to occur via lipid raft-mediated endocytosis negatively regulated by caveolin-1 and to be dependent on intact ERK1/2 signaling [31]. Conversely, a study by Soares et al. indicated the presence of hexameric channels of Connexin 43 (Cx43) in exosomes. These experiments demonstrated colocalization of labeled exosomes and Cx43 protein as well as increased transfer of plasmid DNA with expression of Cx43 in both donor and target cells [32]. These studies demonstrate that exosomal uptake occurs via numerous mechanisms; thus, clinical application of therapeutic exosomes may rely on understanding and exploiting their method of uptake for successful treatment.

## 4. Exosomes in Molecular Signaling

Exosomes have been found in many bodily fluids including blood, urine, and plasma, supporting their role in intercellular communication [33, 34]. Crosstalk between cardiac muscle cells and endothelial cells is critical for regulating cardiac blood vessels to meet the oxygen and nutrient demand of the myocardium. Exosomes have been shown to play a role in communication between cardiac cell types. Vrijsen et al. demonstrated that exosomes from human cardiomyocyte progenitor cells (CPCs) stimulated migration of endothelial cells in a dose-dependent manner using a wound healing assay [35]. Another study by Ong et al. utilized concomitant post-MI injection of mouse CPCs and exosomes from endothelial cells (ECs) overexpressing HIF-1*α*. The transplanted CPCs displayed increased survival in the exosome injected group, as compared to CPC injection alone, demonstrating the cytoprotective properties of exosomes [36]. Additionally, Yu et al. demonstrated that exosomes isolated from conditioned media of GATA4-overexpressing MSCs were enriched in antiapoptotic miRNAs and when injected into rat hearts post-MI mediated cardioprotective effects [37]. Another recent study by Khan et al. demonstrated that mouse embryonic stem cell- (ESC-) derived exosomes have the ability to augment cell survival, proliferation, neovascularization, and cardiac function in the infarcted heart [38]. As proof of principle, Hergenreider et al. utilized ECs transduced with eGFP in a transwell assay with smooth muscle cells (SMCs) to demonstrate transfer of eGFP mRNA to the SMCs [39]. Additional experiments showed miRNA transfer from vesicles isolated from ECs transfected with a miRNA unique to *C. elegans*, when incubated with SMCs [39]. These experiments exhibited exosomal transfer of RNA and miRNA from ECs to SMCs indicating that exosome contents are source cell-specific and can be transferred as paracrine mediators and carriers of genetic information [39]. Overall, these studies demonstrate that various types of cardiac cells signal through exosomes to adapt to changes in their microenvironment.

Exosome secretion from cell cultures has been highly characterized as their isolation from media is mostly straightforward. Unfortunately, characterization of exosome secretion from *in vivo* tissue is not as simple as many possible source cells are present. However, presumed exosome secretion from tissue has been observed using transmission electron microscopy (TEM). In this study, secretion of double membrane-bound vesicles approximately 50 nm in size was visualized in both healthy and ischemic heart tissue [40]. Additional studies in rodent hearts demonstrated exosomes and microvesicle secretion by cardiac progenitor cells [41] and telocytes, an interstitial cell type present in the heart [42]. Altogether, these studies corroborate the secretion of exosomes from the mammalian heart. Moreover, Xiao et al. used CPCs to study whether oxidative stress would affect exosome secretion. In this study, cells were treated with or without H_2_O_2_ and exosome concentrations were measured; results indicated that oxidative stress increased exosome secretion in a dose-dependent manner [43]. These findings further supporting the role of exosomes in signaling in response to stress.

## 5. Exosomes and Cardiac Repair

A growing body of evidence demonstrates that exosomes delivered to cells under oxidative stress stimulate angiogenesis and cytoprotection as well as regulate inflammation and apoptosis [44–46]. Several studies performed using exosomes isolated from various stem cell types investigated their potential role in attenuating cardiac fibrosis and improving cardiac function post-MI (Tables 1 and 2). Source cell type and culture conditions can determine the therapeutic potential of derived exosomes. For instance, ESCs grown under normal conditions produce therapeutic stem cells as observed in a study by Khan et al. which utilized mouse ESC-derived exosomes to treat the heart after acute MI in a mouse model [38]. Results of this study displayed improvement in cardiac function and decreased fibrosis following exosome treatment [38]. Similarly, exosomes isolated from human embryonic stem cell-derived mesenchymal stem cells (ESC-MSCs) cultured under normal conditions also exhibited therapeutic benefit. When these exosomes were used to treat mouse models of MI [47] and ischemia/reperfusion (I/R) injury [48], improvements in cardiac function and decreased infarct size were observed. Furthermore, multiple studies conducted have used bone marrow-derived MSCs (BM-MSCs) as the source cell for exosomes. Bian et al. isolated exosomes from human BM-MSCs and injected them into rat hearts after induction of acute MI. They observed increased blood vessel density along with improved cardiac function in the exosome-treated group, when compared to control [49]. Likewise, work by Shao et al. employed rat BM-MSCs as source cells for exosomes and treatment for a rat model of MI, which also observed increased cardiac function and decreased fibrosis as well as decreased inflammation [50]. Additionally, another study by Feng et al. utilized exosomes derived from BM-MSCs, which were subjected to ischemic preconditioning (IPC) for treating acute MI in mice. This study demonstrated a significant decrease in cardiac fibrosis in mice treated with exosomes from IPC-treated cells, when compared to the non-IPC group [51]. Similarly, Giricz et al. isolated exosomes from rat heart perfusate with and without IPC, used them to treat a rat model of I/R, and showed that infarct size was reduced with IPC-treated exosomes [52]. On the other hand, transplantation of CPCs post-MI is considered to have the greatest potential for cardiac cell therapy due to its capacity to differentiate toward cardiac lineages [53, 54] and augment paracrine effects [9]. To that end, Barile et al. induced acute MI in rats and injected exosomes from human CPCs in the infarct border zone. Increased blood vessel density, decreased scar tissue, and larger areas of viable tissue in the infarct border zone were seen in the exosome-treated group as compared to control [55]. Overall, this study confirmed that exosome signaling stimulated angiogenesis, increased cell survival, decreased apoptosis, and improved cardiac function, when compared to PBS-injected group [55]. In another study by Cambier et al., exosomes from cardiosphere-derived cells (CDCs) were injected into rat hearts in a model of I/R injury and resulted in decreased infarct mass, decreased infiltration of macrophages, and decreased apoptosis of cardiomyocytes [56]. Collectively, these studies demonstrate that exosome treatment improved cardiac function and decreased fibrosis post-MI, which are crucial requirements for future clinical exosome therapy in myocardial repair.

## 6. Exosomes and Angiogenesis

Restoration of blood flow after ischemia-reperfusion (I/R) injury to the infarct region can salvage myocardium by clearing apoptotic cells, decreasing scar tissue, stimulating angiogenesis, and recruiting progenitor cells for tissue regeneration. Stimulating the growth of new microcirculation in the infarct area can help in reviving cardiac function. Angiogenesis is a process of developing new blood vessels from the existing vessels [57]. In stem cell transplantation after MI, the primary beneficial effects are attributable to the paracrine effect of transplanted stem cells and have been shown to stimulate angiogenesis in the infarct and peri-infarct regions of the heart. Under stress or normal conditions, cell-to-cell communication occurs through the secretion of microvesicles or exosomes. Recent studies have demonstrated that exosomes isolated from stem cells such as MSCs, CPCs, CD34^+^ stem cells, and pericardial fluid can stimulate angiogenesis after MI and improve cardiac function [49, 55, 58–68]. MSC-derived exosomes have shown induction of angiogenesis and promotion of proliferation in cellular experiments and animal models with acute MI. Moreover, hypoxia-treated MSC-derived exosomes have shown a greater angiogenic potential, when compared to exosomes from non-hypoxia-treated MSCs [49, 58]. The ischemic treatment in MSCs promotes the expression of several proangiogenic signaling-associated proteins including epithelial growth factor (EGF), fibroblast growth factor (FGF), and platelet-derived growth factor (PDGF). The underlying molecular mechanism of angiogenesis induced by these exosomes is via the activation of NF-*κ*B pathways [58]. The miRNA analysis of these exosomes identified miR-132, miR-30b, miR-30c, miR-424, let-7f, and let-7b-5p to be proangiogenic miRNAs [55, 59–61, 65]. Furthermore, miR-132 from CPC exosomes downregulated RasGAP-p120 enhancing tube formation in endothelial cells [55]. miR-30b from MSC exosomes downregulated DLL4, a negative regulator of vascular sprouting and vessel branching, and thus promoted angiogenesis [55]. The angiogenic effect of let-7b-5p from pericardial fluid-derived exosomes in ECs occurs via inhibition of its target gene, TGFBR1 [59]. MSCs overexpressing Akt, HIF-1*α*, or CXCR4 have exhibited an induction of angiogenesis and improvement of cardiac function after MSC transplantation in acute MI models. Similarly, exosomes derived from Akt, HIF-1*α*, or CXCR4-overexpressed MSCs exhibit tremendous beneficial effects in the treatment of MI via the induction of angiogenesis and the improvement of cardiac function [62–64]. Furthermore, nitric oxide (NO) plays an important role in regulating vascular tone and vascular growth [69–71]. Exosomes derived from NO-stimulated MSCs also show superior angiogenic effects and ameliorated limb function in a murine model of hind limb ischemia [60]. Moreover, the paracrine effect of transplanted human CD34^+^ stem cells exhibits enhanced therapeutic efficacy in ischemic tissue. Exosomes derived from these cells enriched with proangiogenic miRNAs such as miR-126-3p increased angiogenesis of ischemic hind-limb tissue [65].

Overall, angiogenesis is an important process for tissue repair after MI. Recent progress in exosome research provides a new direction in the treatment of ischemic heart disease via the induction of angiogenesis. Continuing investigation of the molecular mechanism of exosomes on angiogenesis will provide a new avenue for the future treatment of MI. However, the uncertainties that need to be addressed are: What is the molecular mechanism used by exosomes in stimulating angiogenesis? What is the best cell type for exosome generation and stimulation of angiogenesis? The further identification of the content of exosomes, including proteins, miRNAs, peptides, and small molecules, will shed the light on the therapeutic value of exosomes in the treatment of MI and its role in the induction of angiogenesis for cardiac repair after MI.

## 7. Exosomes and miRNAs

**Figure 1:**
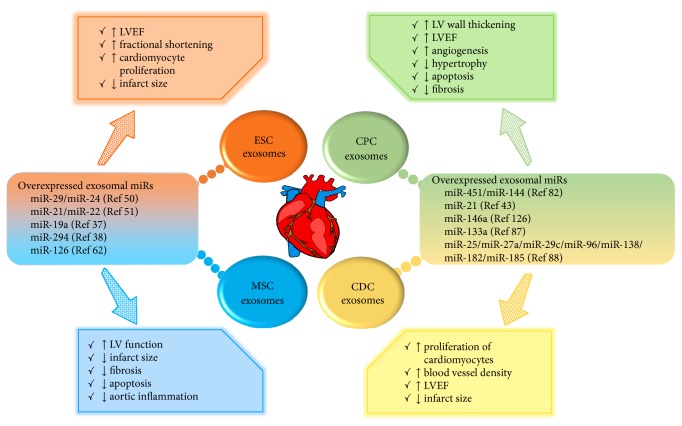
Cardiotherapeutic effects of exosomes isolated from stem cells. Exosomes have been found to play a critical role in cardiac repair. These “nanoshuttles” can impart information to the target cells via miRNAs, which can directly regulate gene expression. Listed are exosomes found to be upregulated in exosomes isolated from various cell lines and the cardioprotective benefits of those exosomes. Exosomes derived from mesenchymal stem cells (MSCs), cardiac progenitor cells (CPCs), embryonic stem cells (ESCs), and cardiosphere-derived cells (CDCs), administered to mice after acute myocardial infarction (AMI) have been shown to enhance cardiac function, angiogenesis, attenuate apoptosis, and fibrosis.

miRNAs are short noncoding RNAs that regulate gene expression at the mRNA level and are estimated to regulate upwards of 30% of human mRNAs [72]. miRNA regulation of gene expression is critical for numerous cellular processes including proliferation, differentiation and apoptosis [73]. Valadi et al. demonstrated the presence of mRNA and miRNA within exosomes and their ability to deliver the cargo to target cells for translation into protein or regulation of gene expression, respectively [74]. Remarkably, the packaged mRNAs are not the result of random association, as microarray analysis revealed that specific mRNA sequences are targeted for packaging into exosomes [74]. Additionally, several recent studies have reported particular miRNAs to be enriched in exosomes [75–77]. Exosomal miRNA can be transferred to target cells where they can actively promote changes in gene expression and respond to stress (Figure 1). ESC-derived exosomes demonstrated significant enrichment of the ESC-specific miR-290 family (including miR-291, miR-29, and miR-295) and these miRNAs were detected in mouse hearts 5 days after MI [38]. Subsequent miR-294 gain-of-function experiments performed in CPCs showed an increase in the number of CPCs in S-phase, and mRNA expression of cyclins also increased as compared to untreated cells [38]. Further molecular studies showed that treatment of CPCs with miR-294 mimic increased AKT phosphorylation, increased protein expression of CPC multipotency marker nucleostemin [78] and pluripotency regulator LIN28 [79], and increased mRNA expression of pluripotency markers c-Myc and Klf4, as compared to miR-291 mimic and untreated controls [38]. Exposure of CPCs to H_2_O_2_ stress resulted in increased proliferation and survival when treated with miR-294 mimic; thus, miR-294 aided in regulating CPC cell cycle and promoted proliferation and survival [38]. Studies by Xiao et al. showed CPCs exposed to oxidative stress secreted exosomes with significantly increased amounts of miR-21 [43], which has been shown to be important in the response to oxidative stress [80]. Cardiomyocytes exposed to H_2_O_2_ display decreased levels of miR-21 expression and increased apoptosis via caspase activation, as compared to unstressed controls [43]. Interestingly, pretreatment of CMs with stressed CPC-derived exosomes (or miR-21 mimic) rescued the decrease of miR-21 and exhibited decreased apoptosis when exposed to oxidative stress [43]. These findings support the antiapoptotic role of miR-21 in cytoprotection, which further studies revealed to occur by directly targeting PDCD4—a promoter of tumor cell apoptosis [81]. Similarly, exosomes isolated from mouse CPCs which demonstrated protection against oxidative stress-induced apoptosis were found to be enriched in miR-451/−144 [82]. Cell-based studies have demonstrated that miR-451 expression is directly regulated by GATA4 [83], a key transcription factor involved in cardiac development and function [84, 85]. Additionally, miR-133a, a muscle-specific miRNA that is highly abundant in the heart [86], is enriched in exosomes secreted by CPCs and confers protective effects [87]. A study by Agarwal et al. investigated the role of miRNAs in the therapeutic effects of CPC-derived exosomes and identified numerous miRNAs of importance known to regulate fibrosis, cardiac hypertrophy, angiogenesis, and apoptosis [88]. Conclusively, the complex role of exosomal miRNAs in cardiac repair is only beginning to be unraveled and requires further study.

## 8. Exosomes from Human-Induced Pluripotent Stem Cell-Derived Cardiomyocytes

**Figure 2:**
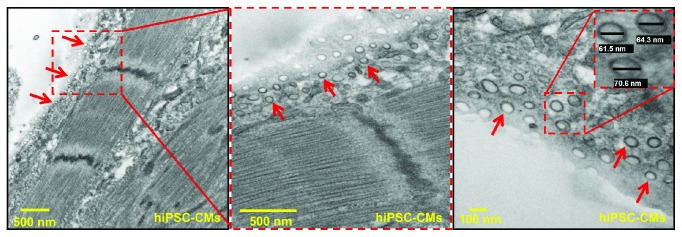
Transmission electron microscopy (TEM) of exosomes isolated from human-induced pluripotent stem cell-derived cardiomyocytes (hiPSC-CMs). Exosomes are indicated by red arrows, and the size of exosomes was measured by ImageJ analysis. The average mean diameter of these exosomes was 66.8 ± 11.5 nm.

Recently, induced pluripotent stem cell-derived cardiomyocytes (hiPSC-CMs) have emerged as a potential source for cardiac regeneration. Previous studies from our lab have demonstrated improved cardiac function and decreased fibrosis following transplantation of hiPSC-CMs post-MI [89, 90]. However, there is a paucity of information about exosomes derived from hiPSC-CMs in cardiac repair. In an effort to understand whether hiPSC-CMs release exosomes under normoxic environment, we have cultured hiPSC-CMs for 2 weeks and performed TEM studies. Our results demonstrate that hiPSC-CMs can secrete exosomes under normoxic microenvironment; these exosomes can be identified in the cytoplasm and close to the periphery of the cell membrane (Figure 2). The size of these exosomes ranged from 46 to 88 nm; studies are in progress in our lab to understand the role of hiPSC-CMs-secreted exosomes in cardiac repair and angiogenesis.

## 9. Exosomes as Diagnostic Markers in Cardiovascular Disease

Exosomes carry cargo representative of the status and microenvironment of their source cells, which changes in response to stressors. Combining this trait with their presence and stability in bodily fluids, exosomes are being heralded as a practical source of diagnostic and prognostic markers. Jansen et al. found that miRNAs present in circulating microvesicles could predict cardiac events. By studying select miRNAs known to regulate vascular performance in patients with stable coronary artery disease, it was discerned that increased expression of miR-126 and miR-199a in circulating microvesicles correlated with a decreased occurrence of a major detrimental cardiovascular event [91]. Moreover, endothelial cells express miR-126, which is involved in angiogenic development and vascular integrity [92] by regulating the cellular response to VEGF signaling [93]. The miR-199 family has an important role in hypoxia-induced cell death via downregulation of HIF-1*α* and stabilization of proapoptotic factor p53 [94]; miR-199a is downregulated in heart failure patients [95]. Additionally, Kuwabara et al. showed that patients with CVD had increased serum levels of miR-1 and miR-133a, indicating myocardial damage [96]. A mouse model of MI demonstrated levels of miR-1 and miR-133a were decreased in the infarcted myocardium and elevated in serum as compared to sham-operated animals, suggesting that these miRs could be an early stage diagnostic marker [96]. Another study by Yang et al. noted that acute MI patients had increased serum exosome levels of miR-30a, which was upregulated by HIF-1*α* during hypoxia and induced autophagy of cardiomyocytes in cellular studies [97]. Furthermore, Matsumoto et al. found that patients with acute MI who developed heart failure have elevated serum exosome levels of miR-192, miR-194, and miR-34a [98], all of which are p53-responsive [99, 100]. In summary, profiling exosomes miRNAs in patients' blood samples post-MI could serve as a reliable repertoire of biomarkers for CVD. This would enable physicians to provide patients the most appropriate treatment when presented in the clinic or to adjust their treatment regimen as needed.

## 10. Exosomes as Cell-Free Therapeutics in Cardiac Repair

Researchers are studying ways to harness the paracrine signaling potential of exosomes in use as a novel cell-free therapeutic. The cardiotherapeutic potential of exosomes was first demonstrated by Brill et al. using human platelet-derived microparticles (PMPs). PMPs were isolated from healthy donors via an ultracentrifugation protocol similar to that utilized for exosome isolation. A rat model of MI was injected with either PMPs or saline immediately after permanent coronary artery ligation. Three weeks post-MI, rats treated with PMPs showed a significant increase in blood vessel density, suggesting the PMPs stimulated angiogenesis in the infarct region [101]. In a study by Vicencio et al. rats were pretreated with a tail vein injection of plasma exosomes isolated from healthy human donors then subjected to acute MI. Results demonstrated reduced infarct size with exosome treatment compared to vehicle control [102]. Additional cell-based experiments showed that pretreatment with human plasma exosomes prior to hypoxia-reoxygenation injury resulted in a significant increase in the population of healthy cells, as well as reducing the population of cells with a low mitochondrial transmembrane potential post-injury [102]. These studies demonstrated the ability of the exosomes to confer resilience to oxidative stress injury.

Specificity of exosome signaling to target cells will allow stimulation of the desired response while decreasing the possibility of off-target effects. Recently, Alvarez-Erviti et al. utilized exosomes that were engineered to target specific cells and deliver their exogenous cargo. This study utilized allogenic dendritic cells transfected to express Lamp2b, an exosomal protein, fused to RVG peptide that specifically binds the acetylcholine receptor [103]. Exosomes isolated from conditioned media were loaded with exogenous siRNA by electroporation and then delivered via tail vein injection into mice. This study exhibited successful and specific delivery of exosomes to neurons, microglia, and oligodendrocytes in the brain without nonspecific tissue delivery. Additionally, gene expression analysis revealed 60% mRNA-level and 64% protein-level knockdown of the siRNA target gene BACE1, a therapeutic target in Alzheimer's disease [103]. Furthermore, preexposure with RVG-exosomes did not attenuate the effect, indicating a lack of immune response elicited by exosomes [103]. Notably, this approach would be successful when the target cells display unique receptors or markers; however, it may also limit the response to a single cell type.

The use of exosomes as a cell-free therapeutic has numerous advantages over stem cell-based transplantation (Table 3). A drawback for stem cell transplantation lies in their potential for teratoma formation [104] and fear of tumorigenicity as cells must pass preclinical *in vivo* tumorigenicity testing in order to receive FDA approval [105–107]. Even with transplantation of highly pure stem cells, teratoma formation is still possible [108] and tumors may develop years after transplantation [109], even with autologous grafting [110]. Conversely, the tumorigenic potential of exosomes is very low as they are a short-term treatment being readily taken up by target cells or flushed out via the blood and urine as demonstrated in studies tracking labeled exosomes [111, 112]. The immunogenicity of exosomes is also minimal, as compared to stem cells [113]. A mouse study by Zhu et al. determined the toxicity and immunogenicity of exosome treatment given over 3 weeks was not significant [114]; however, longer-term studies are needed. Clinical trials in humans using exosomes to treat cancer have shown the treatment to be well-tolerated with only low-level immune responses observed [115–117]. However, it should be noted that these studies were utilizing exosomes in a therapeutic vaccination strategy and aimed to increase the immune response against cancer cells. The major limitation of stem cell therapy for cardiac repair is low survival of transplanted cells in the harsh microenvironment of the ischemic heart. A porcine model of MI treated with MSC injection showed engraftment of 6% of cells to the ischemic myocardium 10 days after treatment [118]. In addition, although cell engraftment is poor in the ischemic heart, improvements in cardiac function are still observed and are credited to the paracrine effects provided by the transplanted cells [8–10], in part mediated by exosomes. Thus, exosome treatment would allow for administration of beneficial paracrine signaling effects without the threat of teratoma formation and very low risk of tumorigenicity and immunogenicity. Ultimately, clinical trial results for stem cell transplantation are inconsistent, with some showing small improvements [119–121] and other showing no improvement in cardiac function [122, 123]. Meanwhile, Tables 1 and 2 illustrate that animal studies of exosome treatment for MI have yielded similar results despite derivation from different source cells. However, successful exosome treatment of chronic and complex conditions in humans will require much more extensive testing and validation to ensure their safety and efficacy.

## 11. Potential Challenges of Exosomes in Cardiac Repair

While exosomes possess great promise as cell-free therapeutics, there are several challenges facing its inception into the clinic. A notable challenge for therapeutic exosomes lies in the optimization and standardization of their isolation. Multiple methods are currently used including differential centrifugation, antibody-based pulldown, size exclusion chromatography, and precipitation with polymers, each with their own advantages and caveats (reviewed in [124, 125]). The appropriate isolation method would deliver sterile exosomes with reproducible purity and potency and would need to be amenable to standards for good manufacturing practices (GMPs). Downstream of isolation, dosage regimens and the route of administration remain debatable as researchers utilize varying conditions in animal models. Researchers are using a wide range of doses (1 *μ*g–30 *μ*g), using different units for determining dose (protein amount or particle number), and delivering the exosomes via varying routes (infusion, intracoronary injection, and intramyocardial injection) (Tables 1 and 2). Frequently, laboratory small animal models are often dosed immediately after induction of MI, which is not practical in the human clinical setting. Conversely, a recent randomized study of acute MI in pigs delivered exosomes 4 weeks after MI, which is more applicable to the clinic [126]. This study also evaluated two methods of delivery of exosomes from human cardiosphere-derived cells and determined intramyocardial injection to be therapeutic and intracoronary infusion to be ineffective, with doses that were extrapolated from small animal models [126]. Endpoint measurements in this study at one month post-treatment showed improved cardiac function, decreased fibrosis, decreased hypertrophy of cardiomyocytes, and increased angiogenesis [126]. Whether these improvements would be maintained long-term is still unknown and needs to be explored. A successful therapeutic would have long-lasting effects without eliciting an immune response that could trigger rejection or clearance. Furthermore, exosome-mediated effects are not based merely on content but are influenced by a multitude of factors, which are not fully understood and off-target effects are possible. For instance, two studies found increased expression of miR-146a in exosomes, one with CPC-derived exosomes and the other with EC-derived exosomes. The CPC-derived exosomes delivered to infarcted mouse hearts inhibited cardiomyocyte apoptosis, enhanced angiogenesis and improved LV ejection fraction [55, 127]. In contrast, the EC-derived exosomes decreased cardiomyocyte metabolic activity in cell-based assays and promoted pregnancy-associated cardiomyopathy in mouse models [128]. Although the studies used different animal models, the differences in outcome demonstrates the need to better understand the effects of using exosomes as a therapeutic in a diverse population.

## 12. Conclusions

Successful cardiac repair post-MI requires tissue regeneration in order to restore cardiac function and decrease LV remodeling. Studies in both *in vitro* and *in vivo* models have demonstrated that exosome contents are dependent on cell type and microenvironment in which the cells are cultured. Furthermore, exosome cargo can be manipulated by altering the genetics and microenvironment in which the stem cells are grown to produce beneficial effects on the target cells. Myriad cell types are present in the heart and coordinated communication amongst them is paramount to efficiently regulate cardiac function. A successful exosome therapeutic regimen for cardiac repair would likely include a combination of exosomes that contain cargo capable of attenuating cardiac fibrosis, inducing angiogenesis and improving cardiac function. Above all, exosomes released by stem cells seem to have several advantages over transplanting stem cells directly into the ischemic heart post-MI. However, further studies are warranted in patients to optimize dosing and route of administration, as well as study the immunogenic response and possible side effects for future clinical application.

## Figures and Tables

**Table 1:** Exosomes isolated from stem cells derived from rodent source and their cardioprotective effects on the heart post-MI.

Source cells (rodents)	Conditions for Exo generation	*In vivo* model	Dose/route of administration	Outcomes	References
ESC	40 h in culture—unclear about serum status	Mouse, MI	10 *μ*g total Exo protein/inj; IM inj into border zone at 2 sites	↑ LVEF ↑ FS ↓ ESD ↓ infarct size	Khan et al. [38]

BM-MSC	O/N glucose starvation, (±)IPC; serum-free, collected after 48 h	Mouse, MI	1 *μ*g total Exo protein; injected along border zone	↓ infarct size with IPC	Feng et al. [51]
	10% Exo-depleted FBS, collected after 48 h; during passage 4	Rat, MI	20 *μ*g total Exo protein into 2 sides along infarct border zone	↑ LVEF ↑ FS ↓ fibrosis ↓ inflammation	Shao et al. [50]
	Overexpression of GATA4, 10% Exo-free FBS, collected after 48 h	Rat, MI	Exo from 4 × 10^6^ cells; IM injection at border zone	↑ LVEF ↑ FS ↓ infarct size	Yu et al. [37]

Rat heart perfusate	(±)IPC	Rat, Langendorff I/R	Perfused prior to 30 min global ischemia, 2 h reperfusion	↓ infarct size with IPC perfusate	Giricz et al. [52]

CPC	2% Exo-depleted FBS, collected after 48 h	Mouse, MI	Half of Exo collected from 5 × 10^5^ cells; IM injection	↓ apoptosis	Chen et al. [82]

CD31^+^ cardiac EC	Overexpression of HIF-1*α*, Exo-depleted serum	Mouse, MI	25 *μ*g total Exo protein ± 1 × 10^6^ CPCs; peri-infarct injection	↑ LVEF ↑ FS ↓ Infarct size ↑ capillary density ↑ survival of transplanted CPCs	Ong et al. [36]

ESC: embryonic stem cell; BM-MSC: bone marrow-derived mesenchymal stem cell; CPC: cardiac progenitor cell; EC: endothelial cell; MI: myocardial infarction; Exo: exosome; inj: injection; IM: intramyocardial; IPC: ischemic preconditioning; LVEF: left ventricular ejection fraction; FS: fractional shortening; ESD: end systolic diameter; O/N: overnight.

**Table 2:** Exosomes isolated from stem cells derived from human source and their cardioprotective effects on the heart post-MI.

Source cells (human)	Conditions for Exo generation	*In vivo* model	Dose/route of administration	Outcomes	References
ESC-MSC	Serum-free, collected after 72 h	Mouse, MI	16 *μ*g/kg total Exo protein; tail vein inj 5 min before reperfusion	↑ LV function ↓ infarct size	Arslan et al. [47]
	Serum-free, collected after 72 h	Mouse, I/R	3 *μ*g total Exo protein; tail vein inj 5 min prior to reperfusion	↓ infarct size	Lai et al. [48]

BM-MSC	Serum-free, collected after 72 h at hypoxia (1% O_2_)	Rat, MI	4 inj 20 *μ*g total Exo protein; IM inj into infarct border zone 30 min after ligation	↑ LVEF, FS, LVSP ↓ LVDEP ↓ infarct size ↑ blood vessel mass	Bian et al. [49]

CDC	Serum-free, collected after 5 days	Rat, I/R	10 *μ*g total Exo protein; injected into LV cavity over 20s with aortic cross clamp 10 min into reperfusion	↓ infarct mass ↓ infiltration of macrophage ↓ apoptosis of cardiomyocytes	Cambier et al. [56]
	Serum-free, collected after 15 days at confluence	Mouse, MI	2.8 × 10^9^ Exo; IM inj at 2 sites in peri-infarct area either immediately or 3 weeks later	↑ LVEF ↓ fibrosis ↓ infarct size (for both dose intervals)	Ibrahim et al. [127]
	Serum-free, collected after 15 days at confluence	Pig, MI	16.5 × 10^11^ Exo in 10 injections; IM injection to infarct area	Maintained LVEF ↓ scar mass ↑ blood vessel density ↑ proliferation of cardiomyocytes	Gallet et al. [126]

CPC	1% HSA, collected after 48 h	Rat, MI	30 *μ*g or 300 *μ*g total Exo protein; IM inj into viable myocardium bordering LV infarct zone at 3 sites	↑ LVEF ↑ in systolic LV wall thickening ↓ fibrosis	Barile et al. [55]

PMPs	Healthy donors, no medication for 2 weeks	Rat, MI	5 *μ*g total Exo protein per injection; 4 inj 2 mm from cyanotic region	↑ functional vascularization	Brill et al. [101]

ESC-MSC: embryonic stem cell-derived mesenchymal stem cell; BM-MSC: bone marrow derived mesenchymal stem cell; CPC: cardiac progenitor cell; CDC: cardiosphere-derived cells; PMP: platelet microparticles; MI: myocardial infarction; I/R: ischemia/reperfusion; Exo: exosome; inj: injection; IM: intramyocardial; IPC: ischemic preconditioning; LV: left ventricular; LVEF: left ventricular ejection fraction; FS: fractional shortening; LVSP: left ventricular systolic pressure; LVDEP: left ventricular end-diastolic pressure; HSA: human serum albumin.

**Table 3:** Comparison of stem cell therapy versus exosomes (cell-free) therapy. Exosomes have several advantages over stem cell transplantation; however, they also have disadvantages for therapeutic applications, which have been outlined in this table.

	References
Cell therapy	
Advantages	
Potential for multilineage differentiation	[4–8]
iPSCs—potential for autologous transplantation	[4]
Disadvantages	
Inconsistent clinical trial results	[114–118]
Low engraftment	[6, 7]
Low direct regeneration	[8]
Risk of formation of benign teratoma	[100, 104]
Fear of tumorigenicity—must pass preclinical *in vivo* tumorigenicity testing to receive FDA approval	[101–103, 105]
Immunogenicity—rejection of allogenic transplants	[109]

Exosomes (cell-free) therapy	
Advantages	
Provides active molecules to target cells—mRNA, miRNA, protein	[23–25, 36]
Contents can be modified via source cell manipulation or external means	[119]
Very low immunogenicity	[99, 110]
Disadvantages	
Laborious and inefficient isolation methods	[119, 120]
Short-term use only, do not regenerate	[14–116]

iPSCs: induced pluripotent stem cells.

## Abbreviations

CVD: Cardiovascular disease
CHD: Coronary heart disease
MI: Myocardial infarction
MSC: Mesenchymal stem cells
CPC: Cardiac progenitor cells
EV: Extracellular vesicles
EC: Endothelial cells
ESC: Embryonic stem cells
SMC: Smooth muscle cells
miRNA: MicroRNA
PF: Pericardial fluid
Exo: Exosomes
TEM: Transmission electron microscopy
MV: Macrovesicles
EGF: Epithelial growth factor
FGF: Fibroblast growth factor
PDGF: Platelet-derived growth factor
NO: Nitric oxide
PMP: Platelet-derived microparticles.
